# The beneficial effects of Rosuvastatin in inhibiting inflammation in sepsis

**DOI:** 10.18632/aging.205937

**Published:** 2024-06-14

**Authors:** Ziming Tang, Zheng Ning, Zexuan Li

**Affiliations:** 1 Department of Emergency, Peking University International Hospital, Beijing 102206, China

**Keywords:** Keywords: Rosuvastatin, sepsis, macrophage, inflammation, LPS

## Abstract

Microbial infection-induced sepsis causes excessive inflammatory response and multiple organ failure. An effective strategy for the treatment of sepsis-related syndromes is still needed. Rosuvastatin, a typical β-hydroxy β-methylglutaryl-CoA reductase inhibitor licensed for reducing the levels of low-density lipoprotein cholesterol in patients with hyperlipidemia, has displayed anti-inflammatory capacity in different types of organs and tissues. However, its effects on the development of sepsis are less reported. Here, we found that the administration of Rosuvastatin reduced the mortality of sepsis mice and prevented body temperature loss. Additionally, it inhibited the production of inflammatory cytokines such as tumor necrosis factor (TNF-α), Interleukin-6 (IL-6), interleukin-1β (IL-1β), and migration inhibitory factor (MIF) in peritoneal lavage supernatants of animals. The increased number of mononuclear cells in the peritoneum of sepsis mice was reduced by Rosuvastatin. Interestingly, it ameliorated lung inflammation and improved the hepatic and renal function in the sepsis animals. Further *in vitro* experiments show that Rosuvastatin inhibited lipopolysaccharide (LPS)-induced production of proinflammatory cytokines in RAW 264.7 macrophages by preventing the activation of nuclear factor kappa-B (NF-κB). Our findings demonstrate that the administration of Rosuvastatin hampered organ dysfunction and mitigated inflammation in a relevant model of sepsis.

## INTRODUCTION

Sepsis is a systemic inflammatory response syndrome caused by microorganisms and is a common complication in severe clinical cases, such as burns, severe trauma, infection, shock, and major surgery. [1]. When microorganisms invade the circulatory system, toxins are released to cause excessive immune response, leading to an imbalance in the host body and causing systemic infection. If the infection is not effectively controlled, patients remain in a state of continuous, excessive response and immunosuppression, ultimately developing into severe sepsis and septic shock, which may even lead to death [2]. Data indicates that approximately 100 million people worldwide develop sepsis each year, with over 25% of these cases resulting in death due to ineffective treatment. [3]. In addition, sepsis has become the main cause of death in non-cardiac patients in intensive care units. In mainland China, one in five intensive care unit (ICU) patients have sepsis, and the mortality rate is 35.5% within 90 days [4]. With the scientific application of antibiotics and progression of surgical and drainage techniques, the cure rate of sepsis has significantly improved to a certain level. However, its complex pathogenesis has not yet been fully revealed, and there is still a lack of effective treatment methods [5].

The occurrence and development of sepsis are closely related to many factors such as inflammatory mediators, bacterial endotoxins, coagulation dysfunction, immunodeficiency, and gene polymorphism [6]. After the pathogen invades the body, it produces toxins, and the body’s defense system is immediately activated. Once the danger signal is received, inflammatory cells are rapidly activated, and the activated cells produce and release a series of cytokines and mediators to cause immune dysfunction and widespread injury. The inflammatory factors include bradykinin, prostaglandins, oxygen-free radicals, leukotrienes, and interleukins [7]. Additionally, migration inhibitory factor (MIF) has been considered a necessary factor for NOD-like receptors family pyrin domain containing 3 (NLRP3) inflammasome formation in sepsis and in systemic lupus erythematosus [8]. IL-6, IL-8, and IL-10 are considered early diagnostic factors of a hyperinflammatory state and organ dysfunction in pediatric sepsis [9]. Gram-negative bacteria are the most common pathogens in sepsis. It has been confirmed that there are many enterobacteria such as *Escherichia coli* and *Pseudomonas aeruginosa* in septic patients [10]. Other typical pathogenic bacteria include *Streptococcus pneumoniae* and *Staphylococcus aureus*. Lipopolysaccharide (LPS), a major glycolipid on the outer membrane of Gram-negative bacteria, can induce the activation of inflammatory cells and promote synthesis and release of inflammatory mediators. Experimental results show that injecting a certain amount of LPS into the body can stimulate the synthesis and release of a large number of inflammatory factors, significantly increase blood factors, produce widespread inflammation, and reduce symptoms after giving inhibitory factor drugs [11]. Regulating LPS-induced inflammation is an important strategy for treating sepsis-related diseases.

Rosuvastatin is a strong lipid-lowering drug mainly used for treating hypercholesterolemia and mixed hyperlipidemia. It inhibits the activity of cholesterol synthase by reducing cholesterol synthesis and reduces blood lipid levels [12]. It has a strong lipid-lowering effect, and promising safety and tolerability, and is widely used in clinical practice [13]. In addition, Rosuvastatin also has antioxidant, and anti-thrombotic effects, and can protect endothelial cells to prevent cardiovascular diseases [14]. It is reported to show inhibitory effects on pulmonary inflammation in hyperlipidemia rats [15]. In addition, it alleviates LPS-induced oxidative stress (OS) and inflammation by inhibiting the NLRP3/Toll-like receptor 4 (TLR4) pathway to reduce myocardial injury [16]. Herein, the influence of Rosuvastatin on LPS-induced tissue damage and inflammation in sepsis mice will be studied to provide potential strategies for clinical sepsis treatment.

## MATERIALS AND METHODS

### Animal experiments

6-weeks-old male C57BL/6 mice (20 ± 2g) were obtained from Aniphe Biolaboratory Inc. (China) and divided into 4 groups: Control, Rosuvastatin, LPS, LPS+Rosuvastatin (15 mice/group). Mice in the control and Rosuvastatin groups were orally dosed with normal saline and 5 mg/kg/day Rosuvastatin for 20 weeks [17], respectively. Animals in the LPS and LPS+Rosuvastatin groups were administered via gavage with normal saline and 5 mg/kg/day Rosuvastatin (Cat#BCP02356, BioChemPartner, Shanghai, China) for 20 weeks, respectively. Subsequently, they were intraperitoneally injected with 10 mg/kg LPS 30 min post the last dosing. The survival in each group and the mean body temperature of each animal were recorded.

### Enzyme-linked immunosorbent assay (ELISA)

Levels of TNF-α (Cat#E-EL-M3063), IL-6 (Cat#E-EL-M0044), IL-1β (Cat#88-7013A-86), and MIF (Cat#E-EL-M0771) in peritoneal lavage supernatants of each animal, as well as levels of TNF-α and IL-6 in the supernatant of macrophages, were detected using commercial ELISA kits (eBioscience, USA). Briefly, specific antibody globulin was diluted with buffered solution to 10 μg/mL, which was added into wells and kept at 4° C overnight. Following the removal of the coating solution, 100 μL of sample was added to each well, and the wells were kept overnight at 4° C. After removing the sample, 100 μL of buffered enzyme-labeled specific antibody solution was added, and it was kept for 2 hours. The antibody was then removed, and 100 μL of substrate was added and kept for 30 minutes. Finally, 50 μL of 2 mol/L H_2_SO_4_ was added to terminate the reaction. The absorbance (D) value at 450 nm was measured using a microplate reader (MD, USA).

### Biochemistry analysis

The cardiac puncture was used to collect blood 24 hours after LPS administration. Then, the levels of oxaloacetic transaminase and creatinine were determined using an automatic biochemical analyzer (Roche, Switzerland).

### The counting of leukocytes

The peritoneal lavage fluid was diluted with 2% acetic acid, and the total number of cells was counted under a light microscope. To classify and count the cells, the sample was centrifuged on a microscopic slide and then stained using the Panoptic fast kit.

### Hematoxylin-eosin (HE) staining

Lung tissues were fixed with 4% paraformaldehyde, followed by paraffin-embedding and slicing. Slides with a thickness of 4 μm were obtained, cleaned and immersed in xylene and ethanol solutions of varying concentrations for 30 minutes. Dehydration was then carried out using anhydrous ethanol followed by Hematoxylin-eosin staining. Finally, the slices were transparentized using xylene and ethanol, sealed with a neutral resin, and randomly selected for observation under a microscope. Ten fields of view were randomly selected for observation and photography, and pathological changes in the tissue were recorded.

### Western blotting

Lung tissues were taken out of liquid nitrogen and naturally thawed. The tissue was then cut into small pieces using surgical scissors and evenly ground in a grinder. RIPA protein extraction solution was used to extract total protein from the tissue or macrophages on ice. After centrifugation at 4° C and 12000r/min for 15min, the supernatant was collected. The protein concentration was measured using the bicinchoninic acid (BCA) method. 20 μg protein per sample was loaded onto a sodium dodecyl sulfate-polyacrylamide gel electrophoresis (SDS-PAGE) gel for electrophoresis separation, transferred to a membrane, and blocked with non-fat milk powder. Then, primary antibody against inducible nitric oxide synthase (iNOS) (1:1000, ab283655, Abcam, USA), Cyclooxygenase 2 (COX-2) (1:2000, ab188184, Abcam, USA), TLR4 (1:2000, Cat#AF7017, Affinity Biosciences, Changzhou, China), p-NF-κB p65 (1:1000, Cat#AN371, Beyotime, Beijing, China), NF-κB p65 (1:2000, Cat#AF0246, Beyotime, Beijing, China), or β-actin (1:5000, Cat#ab8227, Abcam, USA) was added and incubated overnight on a shaker at 4° C. The next day, the primary antibody was recovered, and horseradish peroxidase (HRP)-labeled secondary antibody (1:4000, Cat#ab288151, Abcam, USA) was added. After incubation on a shaker for 2h, electrochemiluminescence (ECL) reagent was used for exposure and development in a dark room. The film was then processed, decolored, and archived. The relative expression level of the protein was calculated by comparing its gray value with β-actin protein’s gray value.

### Cell culture and treatment

RAW 264.7 macrophages were obtained from ATCC (USA) and cultured in Dulbecco’s modified eagle medium (DMEM) supplemented with 10% fetal bovine serum (FBS) under the condition of 5% CO_2_ and 37° C. To study the function of Rosuvastatin *in vitro*, RAW 264.7 macrophages were stimulated with LPS (10 ng/mL) with or without Rosuvastatin (10 and 20 μM) for 24 h.

### Measurement of NF-κB activity

The plasmids, including NF-κB-Luc, pGL3-Basic, and pGL3-Control, were transfected into RAW 264.7 macrophages. After 48 hours of transfection, cells were collected and transferred to 1.5ml EP tubes. Tubes were centrifuged at 1000r/min for 5 minutes, and the supernatant was discarded. 200 μL of reporter gene cell lysis buffer was added to each tube, mixed by shaking for 30 seconds, and incubated for 5 minutes. Tubes were then centrifuged at 12000r/min for 30 seconds, and 20 μL of the supernatant was added to 100 μL of luciferin and mixed immediately. The resulting fluorescence was detected using a fluorimeter (Olis, USA).

### Statistical analysis

Data that followed a normal distribution were expressed as mean ± standard deviation (S.D.) and analyzed using the software SPSS 17.0. Analysis of variance (ANOVA) was used for comparing multiple groups of data, while the least significant difference (LSD) method was employed for pairwise comparisons. Scheffe’s test was used as the post-hoc test. A P-value of less than 0.05 was considered statistically significant.

## RESULTS

### Rosuvastatin reduced mortality and body temperature loss in sepsis mice

**Figure 1:**
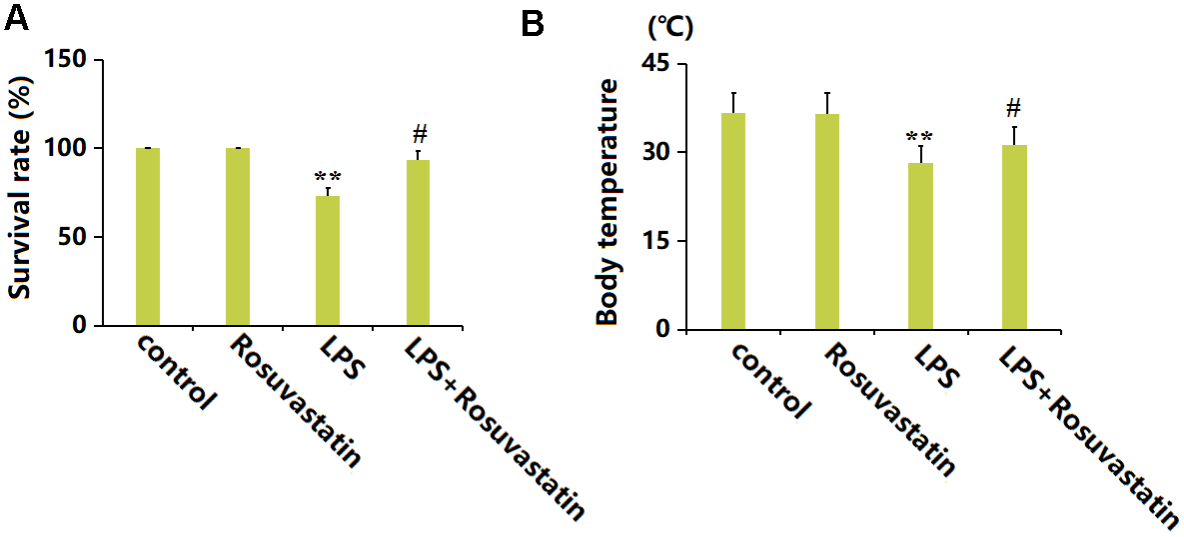
**Rosuvastatin reduced mortality and prevented body temperature loss in sepsis mice.** Mice were divided into four groups: control, Rosuvastatin, LPS, and LPS+Rosuvastatin (15 mice/group). (**A**) Percent survival of experimental mice; (**B**) Body temperature (n=8, **, P<0.01 vs. control group; #, P<0.05 vs. LPS group).

Mice were dosed with 5 mg/kg/day Rosuvastatin for 20 weeks, with or without subsequent LPS stimulation. The survival percentage of experimental mice was maintained at 100% in the Rosuvastatin group but greatly reduced to 73.3% by LPS, then markedly increased to 93.3% in the LPS+ Rosuvastatin group (Figure 1A). Furthermore, the mean body temperatures in the control, Rosuvastatin, LPS, and LPS+ Rosuvastatin groups were 36.6° C, 36.5° C, 28.2° C, and 31.3° C, respectively (Figure 1B).

### Rosuvastatin inhibited the production of inflammatory cytokines in peritoneal lavage supernatants of animals

**Figure 2:**
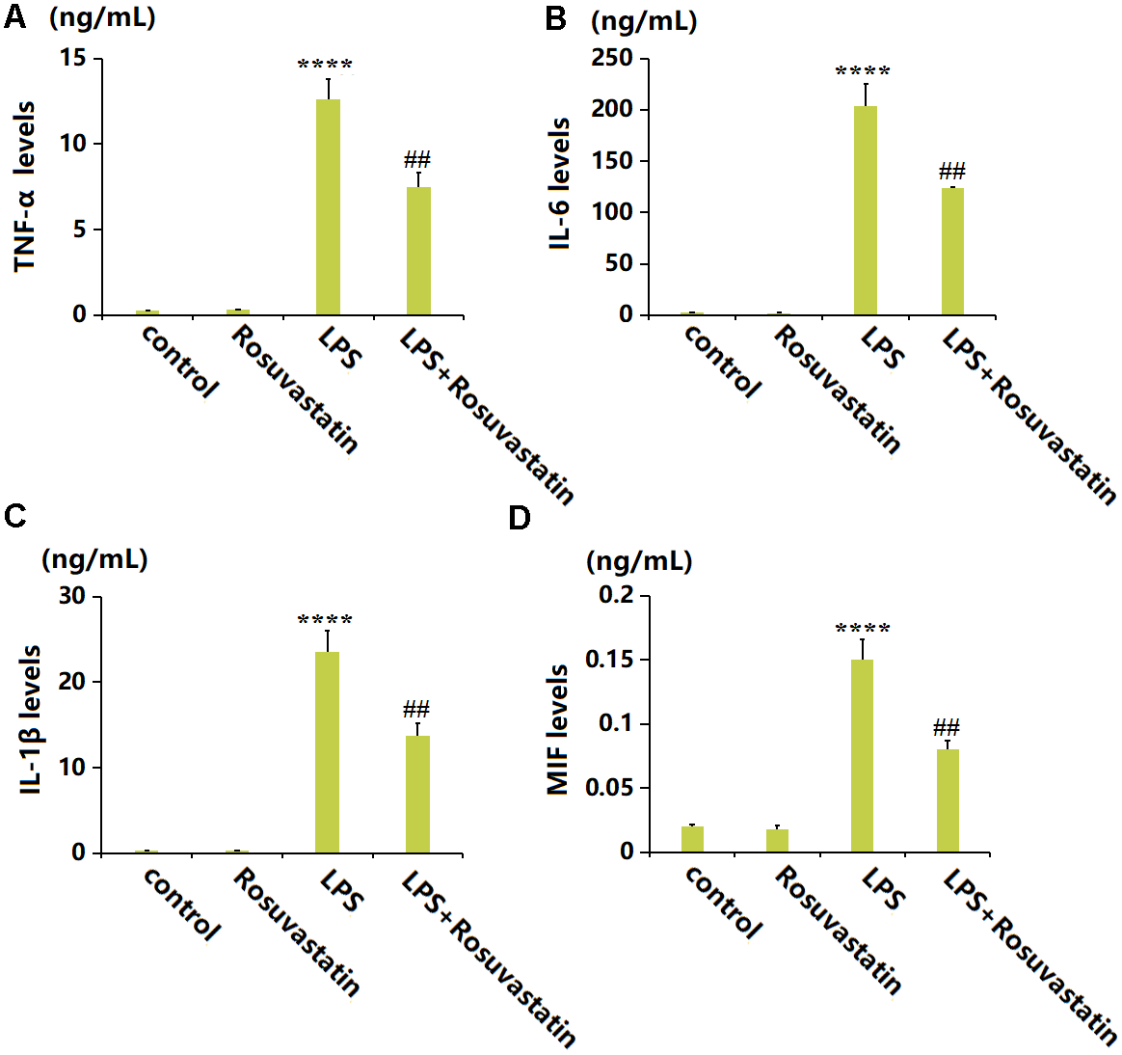
**Rosuvastatin inhibited the production of inflammatory cytokines in peritoneal lavage supernatants of animals.** (**A**) The levels of TNF-α; (**B**) The levels of IL-6; (**C**) The levels of IL-1β; (**D**) The levels of MIF (n=8, ****, P<0.0001 vs. control; ##, P<0.01 vs. LPS group).

To evaluate the inflammatory state in each animal, peritoneal lavage supernatants were collected for detection. The TNF-α level was slightly increased from 0.25 to 0.31 ng/mL by Rosuvastatin and markedly increased to 12.6 ng/mL by LPS stimulation, then greatly reduced to 7.5 ng/mL in the LPS+ Rosuvastatin group (Figure 2A). The IL-6 levels in the control, Rosuvastatin, LPS, and LPS+ Rosuvastatin groups were 2.6, 2.1, 203.5, and 123.7 ng/mL, respectively (Figure 2B). Moreover, the IL-1β level was slightly increased from 0.31 to 0.32 ng/mL by Rosuvastatin and notably increased to 23.5 ng/mL by LPS stimulation, then remarkably declined to 13.7 ng/mL in the LPS+Rosuvastatin group (Figure 2C). The MIF levels in the control, Rosuvastatin, LPS, and LPS+ Rosuvastatin groups were 0.02, 0.018, 0.15, and 0.08 ng/mL, respectively (Figure 2D).

### Rosuvastatin treatment was able to significantly reduce cell migration into the peritoneum

**Figure 3:**
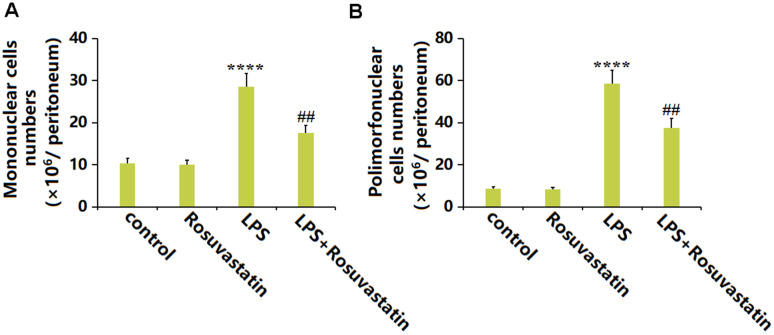
**Rosuvastatin treatment was able to significantly reduce cell migration into the peritoneum.** (**A**) Numbers of mononuclear cells; (**B**) Numbers of polymorphonuclear cells (n=8, ****, P<0.0001 vs. control; ##, P<0.01 vs. LPS group).

Subsequently, the migration of immune cells was evaluated. The number of mononuclear cells in the peritoneum was slightly decreased from 10.3 ×10^6^/peritoneum to 10.1 ×10^6^/peritoneum by Rosuvastatin and significantly increased to 28.6 ×10^6^/peritoneum by LPS stimulation, then markedly reduced to 17.5 ×10^6^/peritoneum in the LPS+ Rosuvastatin group (Figure 3A). Moreover, the number of Polymorphonuclear cells in the control, Rosuvastatin, LPS, and LPS+ Rosuvastatin groups was 8.6, 8.3, 58.5, and 37.6 ×10^6^/ peritoneum, respectively (Figure 3B).

### Rosuvastatin reduced lung inflammation in LPS-treated mice

**Figure 4:**
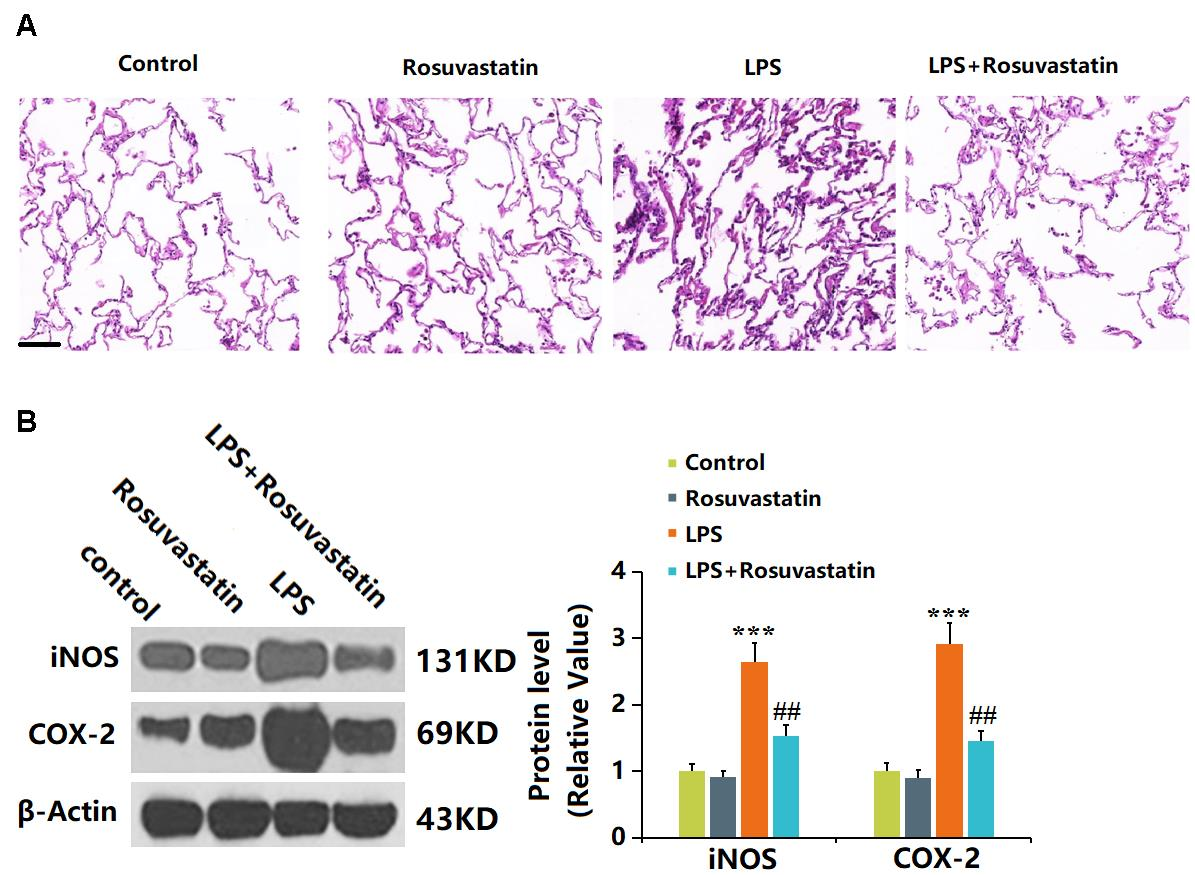
**Rosuvastatin reduced lung inflammation in LPS-treated mice.** (**A**) Lung tissues from each treated group were fixed, sectioned, and stained with hematoxylin and eosin (H and E staining); Scale bar, 500 μm; (**B**) The expression of iNOS and COX-2 proteins in lung tissues was detected using Western blotting (n=8, ***, P<0.005 vs. control; ##, P<0.01 vs. LPS group).

Lung tissue is severely influenced by sepsis [18]. No significant pathological changes in lung tissues were observed in the control and Rosuvastatin groups. In the LPS group, the infiltration of immune cells in the lung tissue was increased, the necrosis severe, and hyperemia and edema observed, which were sharply alleviated in the LPS+ Rosuvastatin group (Figure 4A). Furthermore, iNOS and COX-2 levels in lung tissues were slightly reduced in the Rosuvastatin group and notably increased by LPS stimulation, then markedly reduced by Rosuvastatin (Figure 4B).

### Rosuvastatin improved the hepatic and renal function in sepsis animals

**Figure 5:**
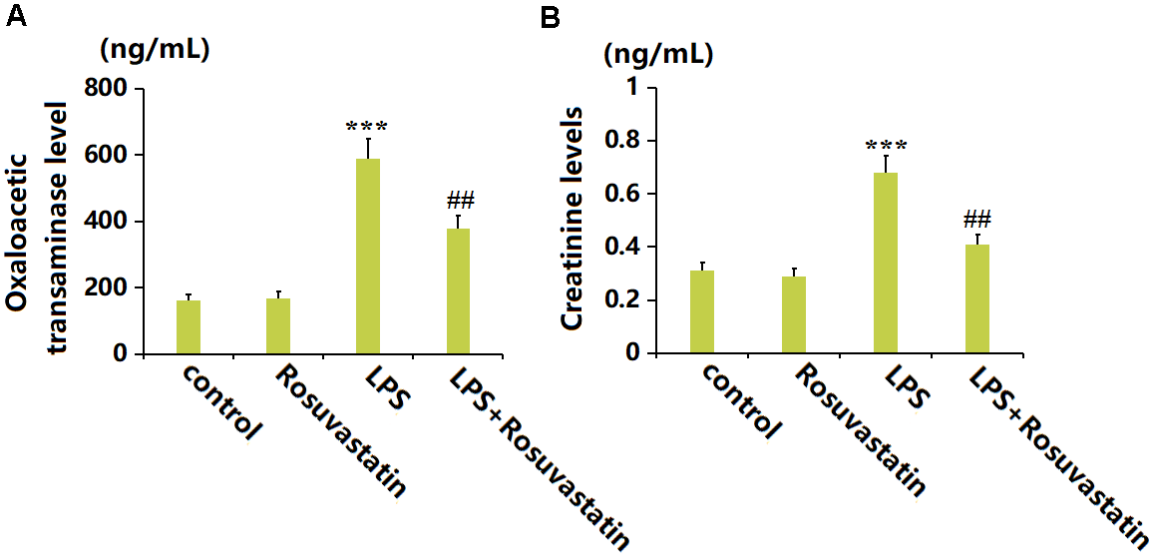
**Rosuvastatin improved the hepatic and renal function in the sepsis animals.** (**A**) The levels of Oxaloacetic transaminase; (**B**) The levels of Creatinine (n=8, ***, P<0.005 vs. control; ##, P<0.01 vs. LPS group).

Heart and kidney function is reported to be severely impaired by sepsis [19, 20]. The oxaloacetic transaminase level was slightly changed in the Rosuvastatin group and greatly elevated to 588.2 ng/mL by LPS stimulation, then remarkably decreased to 379.5 ng/mL by Rosuvastatin (Figure 5A). Moreover, the Creatinine levels in the control, Rosuvastatin, LPS, and LPS+ Rosuvastatin groups were 0.31, 0.29, 0.68, and 0.41 ng/mL, respectively (Figure 5B).

### Rosuvastatin inhibited LPS-induced production of proinflammatory cytokines in RAW 264.7 macrophages

**Figure 6:**
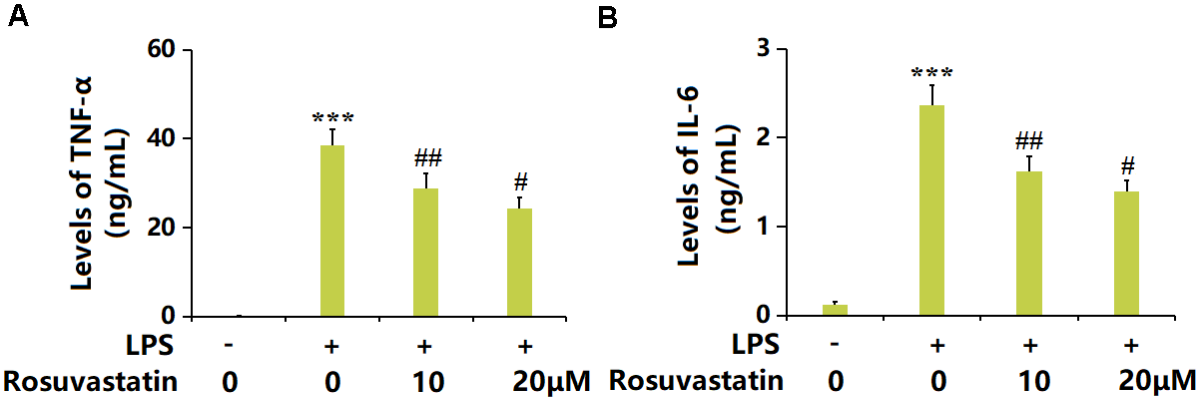
**Rosuvastatin inhibited LPS-induced production of proinflammatory cytokines in RAW 264.7 macrophages.** Cells were stimulated with LPS (10 ng/mL) with or without Rosuvastatin (10 and 20 μM) for 24 hours. (**A**) Levels of TNF-α; (**B**) Levels of IL-6 (n=8, ***, P<0.005 vs. control; #, ##, P<0.05, 0.01 vs. LPS group).

Macrophages are important effector cells regulating the inflammation in sepsis [21]. RAW 264.7 macrophages were stimulated with LPS (10 ng/mL) with or without Rosuvastatin (10 and 20 μM) for 24 hours. The TNF-α level was greatly increased from 0.23 to 38.5 ng/mL by LPS but markedly decreased to 28.9 and 24.2 ng/mL by 10 and 20 μM Rosuvastatin, respectively (Figure 6A). Moreover, the IL-6 levels in the control, LPS, 10 μM Rosuvastatin, and 20 μM Rosuvastatin groups were 0.12, 2.36, 1.62, and 1.39 ng/mL, respectively (Figure 6B).

Rosuvastatin inhibited LPS-induced activation of NF-κB in RAW 264.7 macrophages.

**Figure 7:**
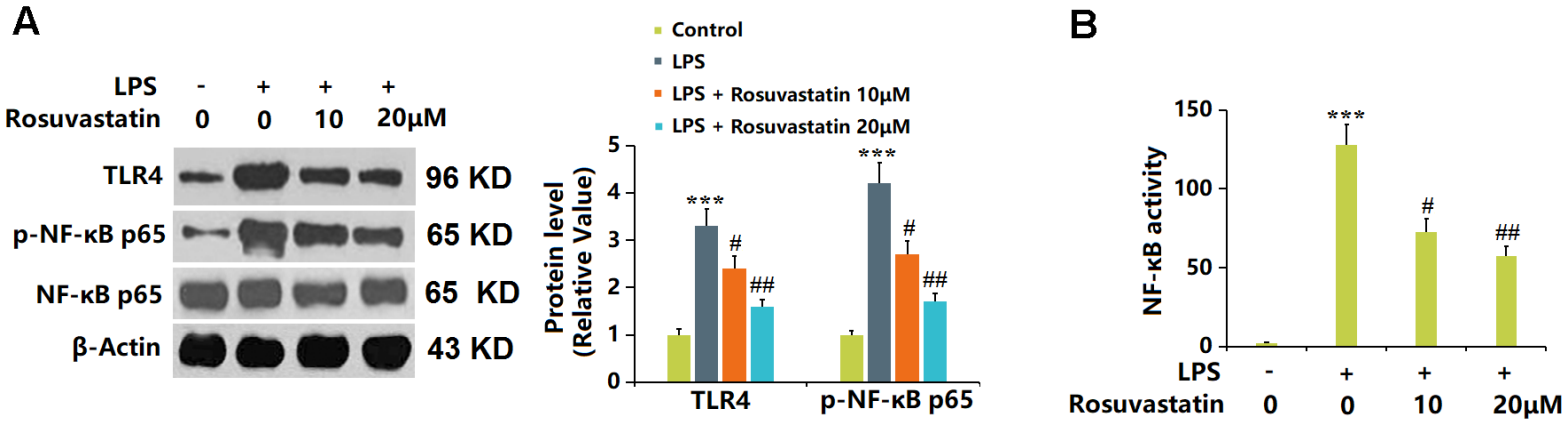
**Rosuvastatin inhibited LPS-induced activation of NF-κB in RAW 264.7 macrophages.** Cells were stimulated with LPS (10 ng/mL) with or without Rosuvastatin (10 and 20 μM) for 24 hours. (**A**) The levels of TLR4 and p-NF-κB p65 were measured by Western blot analysis; (**B**) NF-κB activity was measured using luciferase assays (n=8, ***, P<0.005 vs. control; #, ##, P<0.05, 0.01 vs. LPS group).

TLR4 and p-NF-κB p65 were notably upregulated by LPS in macrophages, then greatly downregulated by 10 and 20 μM Rosuvastatin (Figure 7A). Moreover, in the measurement of NF-κB activity, the markedly increased fluorescence intensity observed in LPS-stimulated macrophages was notably reduced by 10 and 20 μM Rosuvastatin (Figure 7B).

## DISCUSSION

The development of sepsis is often accompanied by multi-organ dysfunction, involving a variety of pathophysiological processes such as imbalance of inflammatory response, immune disturbance, mitochondrial damage, coagulation dysfunction, endoplasmic reticulum stress, and autophagy, which ultimately lead to organ failure [22]. Once these pathogens invade the body, they multiply and cause early inflammatory response imbalance, developing into sepsis. As one of the most common factors, bacteria can activate the innate immune system, promote phagocytosis of macrophages to pathogens, and produce a series of inflammatory factors to trigger cell hormone storms [23]. Uncontrolled inflammation participates in sepsis, and early effective inhibition of inflammation is of great significance [24]. Herein, in line with results claimed by Li [25], Sun [26], and Jiao [27], multi-organ injury, including hepatic, renal, and pulmonary damage, was observed in sepsis mice, all of which were sharply alleviated by Rosuvastatin, implying its protective function against tissue injury induced by sepsis. Moreover, similar to data presented by Oliveira [28], severe inflammation in peritoneal lavage supernatants and enhanced migration of both mononuclear and polymorphonuclear cells into the peritoneum were observed in sepsis mice, but markedly prevented by Rosuvastatin, suggesting its suppressive effect against the activation of immune inflammation during sepsis.

Macrophages are extremely important immune cells in the body’s immune barrier, developed from bone marrow cells and differentiated into monocytes through the circulation to various organs and tissues. They are primarily responsible for participating in local inflammatory reactions [29]. The immune system can defend against infection and external damage by promoting inflammation. Under normal circumstances, inflammation is a life-saving response, and individuals with inherited defects in inflammatory components are more susceptible to infection [30]. However, uncontrolled inflammation can be harmful to the body and may lead to excessive tissue damage. In some diseases, inflammation may cause more damage than the infection itself [31]. As members of the innate immune response, macrophages participate in identifying and phagocytizing foreign substances, killing pathogens, and releasing inflammatory mediators [32]. In the early stages of bacterial infection, macrophages mainly function to recognize and phagocytize pathogens to eliminate bacteria and release inflammatory mediators. When the inflammatory response further strengthens, macrophages are highly activated and release cytokines that promote inflammation, clinically termed a “cytokine storm”. Tissue damage, such as to the lungs, kidneys, and liver, is then induced to result in multiple organ dysfunction. In the late stages of sepsis, the body’s anti-inflammatory response predominates and there is a long-term alternation between pro-inflammatory and anti-inflammatory responses, which mutually reinforce each other, resulting in immune dysfunction, accompanied by an increase in organ damage and even organ failure, as well as immune cell exhaustion, further leading to septic shock and patient death [33]. Therefore, regulating macrophage function and inflammatory cytokine secretion in the early stages of infection is key to preventing the further development of sepsis. Herein, the *in vitro* experiments revealed that the inflammatory state of RAW 264.7 macrophages was markedly repressed by Rosuvastatin, implying that the protection by Rosuvastatin against sepsis might be correlated to its inhibition of the activation of macrophages.

Macrophages mainly function through receptors on their surface that recognize pathogen/damage-associated molecular patterns (PAMP/DAMP). TLR4 on the surface of macrophages is a member of the Toll family and can recognize evolutionarily conserved pro-inflammatory components in microorganisms [34]. In the process of bacterial infection, the main component of the outer membrane of Gram-negative bacteria, LPS, can activate TLR4, triggering pro-inflammatory response transmission and promoting the body’s clearance of bacteria [35]. The activation of TLR4 and its downstream signaling pathway plays a crucial role in the development of inflammation. With the help of LPS-binding protein (LBP), CD14, and MD-2, TLR4 binds to LPS and forms a dimer, activating TLR4 signaling [36]. The inflammation induced by macrophages is largely mediated by NF-κB signaling [37]. The signal is activated through either MyD88-dependent or -independent pathways to activate NF-κB, promoting NF-κB p65 to enter the nucleus and bind to specific sequences in DNA, initiating downstream transcription and translation of pro-inflammatory cytokines [38]. Herein, activation of the TLR4/NF-κB axis in LPS-stimulated RAW 264.7 macrophages was sharply repressed by Rosuvastatin, implying that the suppressed NF-κB signaling might be responsible for the inhibition by Rosuvastatin on the activity of macrophages during sepsis. In future work, co-administering Rosuvastatin and the TLR4/NF-κB axis agonist into the sepsis mice will be performed to identify the protection mechanism of Rosuvastatin against sepsis involving the TLR4/NF-κB axis.

To sum up, Rosuvastatin alleviated sepsis-induced organ dysfunction and inflammation in mice by inhibiting NF-κB signaling. Our data explore the novel pharmacological activity of Rosuvastatin and suggest that it may be further developed as a pharmaceutical agent for the treatment of sepsis. However, there are still several limitations to the current study. Firstly, our conclusions are based on *in vivo* animal models and *in vitro* cell culture studies, and it is difficult to directly extrapolate these conclusions to humans. Secondly, the pathophysiological mechanism of sepsis is complex and needs to be clarified. A wide range of risk factors have been reported to participate in the initiation and development of sepsis. Another limitation of the current study is that the molecular mechanisms whereby Rosuvastatin alleviated sepsis are still unknown. To address these limitations, clinical trials in humans would be critical in realizing the full potential of Rosuvastatin in the treatment of sepsis in the future.
